# A Method for Simultaneously Measuring 6DOF Geometric Motion Errors of Linear and Rotary Axes Using Lasers

**DOI:** 10.3390/s19081764

**Published:** 2019-04-12

**Authors:** Fajia Zheng, Qibo Feng, Bin Zhang, Jiakun Li

**Affiliations:** Key Lab of Luminescence and Optical Information, Ministry of Education, Beijing Jiaotong University, Beijing 100044, China; 16118441@bjtu.edu.cn (F.Z.); bzhang@bjtu.edu.cn (B.Z.); jkli@bjtu.edu.cn (J.L.)

**Keywords:** 6DOF geometric motion errors, measurement, linear axis, rotary axis, CNC machine tools

## Abstract

A novel method for simultaneously directly measuring six-degrees-of-freedom (6DOF) geometric motion errors of CNC machine tools was proposed, and a corresponding measurement system was developed. This method can not only be applied for measuring a linear axis, but also for a rotary axis. A single-mode fiber was used to separate the measuring unit from the laser source in order to ensure system thermal stability and measurement accuracy. The method has the advantages of high efficiency and good accuracy, and requires no complicated decoupling calculation. The positioning error of the linear axis and radial motion error of the rotary axis are measured by laser interferometry and other 5DOF geometric motion errors by laser collimation. A series of experiments were performed to verify the feasibility and effectiveness of the developed measurement system.

## 1. Introduction

The linear and rotary axes are the key components in many mechanical machines, especially for computer numerical control (CNC) machine tools. The linear axis is the basis for linear motion, and the rotary axis is the basis for angular motion. They play a critical role in machining accuracy of machine tools. It has been verified that the machining accuracy can be greatly improved by error compensation [1,2,3]. However, how to obtain the various geometric motion errors of machine tools through measurements is a prerequisite for error compensation. Thus, there is increasing demand for high-precision and high-efficiency measurements of the geometric motion errors of both linear and rotary axes.

For a five-axis CNC machine tool, 42 geometric motion errors need to be measured [4]. To measure these errors, many methods have been proposed, which can be classified into two categories: direct and indirect measurement methods. Direct measurement method has the advantages of high precision, being able to determine the origin of the error without decoupling. In addition, the measurement data can be directly used in the error compensation of machine tools. However, in some cases, measurement efficiency is very low. For example, the laser interferometer can measure only one error at a time; it requires several hours or even several days to conduct a complete test for the three linear axes of machine tools according to ISO 230-1 [5,6,7]. In order to enhance the measurement efficiency, simultaneous measurement of 6DOF geometric motion errors of both linear and rotary axes is an inevitable trend. Various methods for simultaneously measuring 6DOF geometric motion errors of the linear axis have been proposed by researchers, mainly including the collimation [8] and interferometry methods [9,10], as well as combinations of the two [11,12,13,14]. For example, Chen et al. proposed a simple method for simultaneously measuring 6DOF geometric motion errors of a linear axis that can only move 6 mm [8]. Some companies, such as Renishaw and API, have developed the measurement system to measure 6DOF geometric motion errors of the linear axis. The multiaxis laser interferometer systems from JEANer Meßtechnik GmbH and Zygo can measure multi-DOF geometric motion errors for multiple linear axes with high precision and efficiency, but these measurement systems cannot simultaneously measure 6DOF geometric motion errors of the linear axis [15,16]. Furthermore, all these methods and instruments cannot be used to simultaneously measure 6DOF geometric motion errors of the rotary axis. Chen et al. [17] and Park et al. [18] have proposed methods for simultaneously measuring 6DOF geometric motion errors of the rotary axis. To the best of our knowledge, there is no instrument for directly simultaneously measuring 6DOF geometric motion errors of a rotary axis. There are many kinds of the indirect measurement methods, such as ball bar tests, R test, probing of artifacts, tracking interferometer, and machining tests [19,20,21,22,23,24,25]. These methods can be used to measure geometric motion errors of machine tools, and have the advantages of high measurement efficiency and simple measurement configuration. For example, Holub et al. used a self-tracking interferometer in a small three-axis machining center to get all 21 geometric motion errors, and its measurement time was 33 min [19]. Generally speaking, indirect measurement methods have some common disadvantages of requiring complex decoupling and limited measurement range, which make it difficult to conduct an online compensation [22]. 

Laser interferometry is the most effective and precise method for measuring length with a large measurement range, while laser collimation is the right choice for measuring microangles. Based on a combination of the two, we proposed a series of methods for simultaneously measuring 6DOF geometric motion errors of a linear axis [26,27,28]. Based on years of research experience on simultaneously measuring 6DOF geometric motion errors of a linear axis, we began to study the simultaneous measurements of 6DOF geometric motion errors of a rotary axis. Some methods are similar for 6DOF geometric motion error measurements between the linear axis and the rotary axis; the main difference is that the length needs to be measured in a large range for a linear axis and the angle of rotation needs to be measured in a range of 360 degrees for a rotary axis. In addition, servo tracking technology is required for rotary axis measurement in order to keep laser measurement beam back to the measurement unit. Thus, we presented a method for simultaneously and directly measuring 5DOF geometric motion errors of a rotary axis [29]. Based on our previous research works, in this paper, we firstly proposed a method for simultaneously directly measuring 6DOF geometric motion errors of both linear and rotary axes, and developed an integrated system that can be used not only for the linear axis, but also for the rotary axis measurements, thus paving a way to directly measuring all 42 geometric motion errors of a five-axis CNC machine tool with high efficiency. Definitions of 6DOF geometric motion errors of a linear and rotary axis are introduced in Section 2. The proposed method and measurement principles are introduced in detail in Section 3 and Section 4. In Section 5, experiments are performed to demonstrate the feasibility and effectiveness of the developed measurement system.

## 2. 6DOF Geometric Motion Errors of a Linear and Rotary Axis

**Figure 1:**
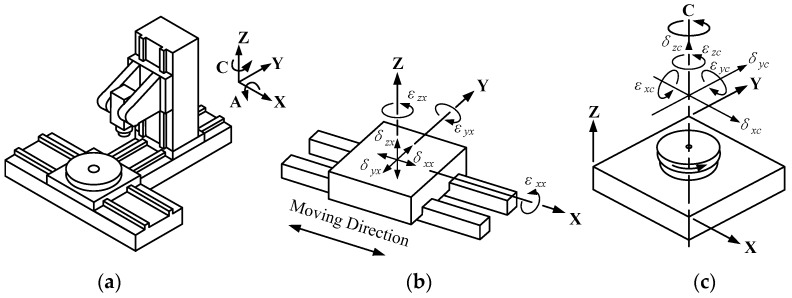
Error model of a five-axis computer numerical control (CNC) machine tool: (**a**) Five-axis CNC machine tool; (**b**) Error model of a linear axis (X); (**c**) Error model of a rotary axis (C).

As shown in Figure 1a, a typical five-axis CNC machine tool is composed of three linear axes (X, Y, and Z) and two rotary axes (A and C). Each axis inherently exhibits 6DOF geometric motion errors that are three translational errors and three angular errors. The 6DOF geometric motion error models for the linear axis and the rotary axis are illustrated in Figure 1b,c, respectively [5].

Table 1 lists 6DOF geometric motion error definitions of the linear axis X and the rotary axis C, respectively.

There are 42 geometric motion errors for a five-axis CNC machine tool to be measured, including 21 errors for the three linear axes, five errors for the spindle, and 16 errors for the two rotary axes.

## 3. Simultaneous Measurement Method for Linear- and Rotary-Axis 6DOF Geometric Motion Errors

### 3.1. Measurement System Configuration

**Figure 2:**
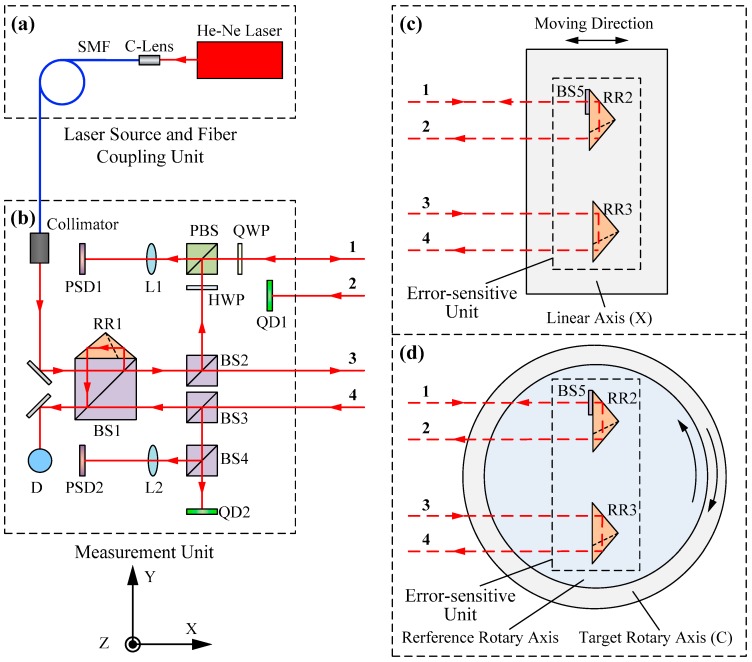
Schematic diagram for simultaneously measuring 6DOF geometric motion errors of both linear and rotary axes; (**a**) Laser source and fiber coupling unit; (**b**) Measurement unit; (**c**) Error-sensitive unit for measuring a linear axis; (**d**) Error-sensitive unit for measuring a rotary axis.

Figure 2 illustrates a schematic diagram of the proposed method, which is composed of three units: a laser source and fiber coupling unit, a measuring unit, and an error-sensitive unit.

As shown in Figure 2a, the laser source and fiber coupling unit consists of a He-Ne laser, a coupling lens (C-Lens), and a single-mode fiber (SMF). The laser beam emitted from the He-Ne laser is coupled into the SMF for transmission. The SMF separates the light source from the measurement unit, thus isolating the heat of the laser and improving the thermal stability of the system. Moreover, the high-quality laser beam emitted from the SMF can be used as the datum for collimation measurements.

In Figure 2b, the beam from the SMF is transmitted to the measurement unit and collimated by a collimator. The collimated beam is split successively by two beam splitters, BS1 and BS2. The beams from BS2 are called beam 1 and beam 3. Beam 1 that is reflected by BS2 travels through a half-wave plate (HWP) and is reflected by a polarization beam splitter (PBS); it then passes through a quarter-wave plate (QWP) and leaves the measurement unit. The transmitted beam 3 from BS2 is parallel to beam 1, and these two beams are transmitted to the error-sensitive unit as the measuring beams. A photodetector (D), two quadrant detectors (QD1 and QD2), and two position-sensitive detectors (PSD1 and PSD2) receive the beams backward-reflected by the error-sensitive unit and perform photoelectric conversions. 

As shown in Figure 2c,d, the error-sensitive unit is composed of two cube corner reflectors (RR2 and RR3) and a beam splitter (BS5). It is small, lightweight, and has no cable connection. Here, beam 1 is split into two parts by BS5, and the transmitted beam from BS5 is reflected by RR2 where it becomes beam 2. Similarly, beam 3 is reflected by RR3 and becomes beam 4. The beam reflected by BS5, and beams 2 and 4 return to the measurement unit with the information of 6DOF geometric motion errors of the linear and rotary axes. In the following section, the measurement principle for each single geometric motion error is described in detail.

### 3.2. Measuring 6DOF Geometric Motion Errors of the Linear Axis

Figure 2a–c illustrates the optical configuration for simultaneously measuring 6DOF geometric motion errors of the linear axis. As shown in Figure 2c, the error-sensitive unit is mounted on a linear axis (e.g., X) and moves along with it.

In Figure 2b, beam 1 is employed to measure yaw and pitch and beam 3 is used to measure the positioning and straightness errors, and these two beams are the datum for measuring the roll error.

### 3.3. Measuring 6DOF Geometric Motion Errors of the Rotary Axis

The configuration for measuring 6DOF geometric motion errors of the rotary axis is illustrated in Figure 2a,b,d. The beams reflected by the error-sensitive unit can move out of the measurement unit if the target rotary axis (e.g., C) rotates at a large angle. To address this problem, there is a precision reference rotary axis, which is mounted coaxially with the target rotary axis. In fact, the purpose of using the reference rotary axis is to keep the beam reflected by BS5 focused at the center of PSD1. Thus, when the target rotary axis rotates clockwise, the reference rotary axis is driven to rotate inversely and synchronously by using servo tracking technology, and its rotation angle can be measured directly by a circular grating. In this manner, the interferometric beam 4 for measuring the radial motion error in the X-axis direction is not interrupted, and the measurement range of the angular positioning error can reach 360 degrees. 

In Figure 2b, beam 1 is used to measure the angular positioning error and tilt motion error around the Y-axis, and beam 3 is employed to measure the radial and axial motion errors; they are the datum for measuring the tilt motion error around the X-axis.

## 4. Measurement Principle for Each Single Geometric Motion Error

**Figure 3:**
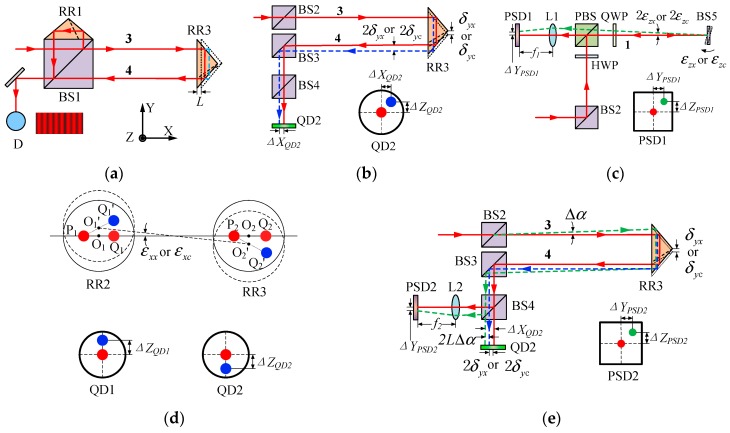
Schematic diagram for measuring each single geometric motion error: (**a**) Linear error in X-axis direction; (**b**) Linear errors in Y- and Z-axis directions; (**c**) Angular errors around Y- and Z-axis; (**d**) Angular error around X-axis; (**e**) Laser beam drift compensation.

According to the principle for simultaneously measuring 6DOF geometric motion errors of both linear and rotary axes shown in Figure 2, the measurement principle for each single geometric motion error is shown in Figure 3. Below are the details of these single error measurements.

### 4.1. Positioning Error of the Linear Axis and Radial Motion Error in the X-Axis Direction of the Rotary Axis

Measurements of the positioning error of the linear axis and radial motion error in the X-axis direction of the rotary axis are based on laser interferometry. As shown in Figure 3a, the beam reflected from BS1 serves as the reference beam, while beam 3 is used as the measuring beam, and the interference signal can be detected by the detector D. The positioning error $\delta_{xx}$ of the linear axis can be obtained as

(1a) $$\delta_{xx}=L-L’$$

The radial motion error $\delta_{xc}$ in the X-axis direction of the rotary axis is given as

(1b) $$\delta_{xc}=L$$

where L is the moving displacement of RR3 for the linear axis or the displacement change for the rotary axis, which is obtained by laser interferometry; L’ denotes the nominal displacement of the linear axis that is given by the machine tool.

### 4.2. Straightness Errors of the Linear Axis, Radial Motion Error in the Y-Axis Direction, and Axial Motion Error of the Rotary Axis

RR3 is the error-sensitive element for straightness errors of the linear axis, and radial motion error in the Y-axis direction and axial motion error of the rotary axis. As shown in Figure 3b, if there are straightness errors in Y- and Z-axis directions of the linear axis, the position changes of beam 4 on the detector QD2 are double. Thus, the straightness errors ($\delta_{yx}$, $\delta_{zx}$) in the Y- and Z-axis directions of the linear axis can be obtained as

(2a) $$\begin{array}{l} \delta_{yx}=\frac{\Delta X_{QD2}}{2}\\ \delta_{zx}=\frac{\Delta Z_{QD2}}{2} \end{array}$$

where $\Delta X_{QD2}$ and $\Delta Z_{QD2}$ denote the positional changes of light spot on QD2 in the X- and Z-axis direction, respectively. Both of them can be obtained by the system.

Similarly, the radial motion error $\delta_{yc}$ in the Y-axis direction and axial motion error $\delta_{zc}$ of the rotary axis can be expressed as

(2b) $$\begin{array}{l} \delta_{yc}=\frac{\Delta X_{QD2}}{2}\\ \delta_{zc}=\frac{\Delta Z_{QD2}}{2} \end{array}$$

### 4.3. Yaw and Pitch of the Linear Axis, Angular Positioning Error, and tilt Motion error Around the Y-Axis of the Rotary Axis

BS5 is used as the error-sensitive element for the yaw and pitch of the linear axis, and angular positioning error and tilt motion error around the Y-axis of the rotary axis. As shown in Figure 3c, the beam reflected by BS5 transmits through a QWP and PBS and is focused on PSD1 by lens L1. According to the autocollimation measurement principle, the yaw $\varepsilon_{zx}$ and pitch $\varepsilon_{yx}$ of the linear axis can be expressed as

(3a) $$\begin{array}{l} \varepsilon_{zx}=\frac{\Delta Y_{PSD1}}{2f_{1}}\\ \varepsilon_{yx}=\frac{\Delta Z_{PSD1}}{2f_{1}} \end{array}$$

where $\Delta Y_{PSD1}$ and $\Delta Z_{PSD1}$ are positional changes of light spot on PSD1 in the Y- and Z-axis direction, respectively. Both of them can be obtained by the system. $f_{1}$ is the focal length of L1 and it is a constant.

For a rotary axis measurement, theoretically, if the reverse rotation angle of the reference axis is the same as the rotation angle of the axis under test, the reading in the Y-axis direction on the detector PSD1 is zero. In fact, the reverse rotation angle of the reference axis is different from the rotation angle of the axis under test because of the angular positioning error of the rotary axis, and the angular positioning error $\varepsilon_{zc}$ as well as tilt motion error $\varepsilon_{yc}$ around the Y-axis can be expressed as

(3b) $$\begin{array}{l} \varepsilon_{zc}=\frac{\Delta Y_{PSD1}}{2f_{1}}+\theta_{ref}-\theta \\ \varepsilon_{yc}=\frac{\Delta Z_{PSD1}}{2f_{1}} \end{array}$$

Here, $\theta_{ref}$ denotes the rotation angle of the reference axis that is measured by the circular grating from the system; *θ* is the nominal rotation angle of the rotary axis given by the machine tool.

### 4.4. Roll of the Linear Axis and tilt Motion Error Around the X-Axis of the Rotary Axis

RR2 and RR3 are the error-sensitive components for measuring the roll of the linear axis and tilt motion error around the X-axis of the rotary axis. As shown in Figure 3d, P_1_ and P_2_ are the incident spots of the beams 1 and 3 on RR2 and RR3, respectively; Q_1_ and Q_2_ are the emergent spots of the reflected light; O_1_ and O_2_ are the centers of the incident planes of RR2 and RR3, respectively. Q_1_ and Q_2_ are in the same position in Z-axis direction without roll error. If there is a roll error, the positions of Q_1_ and Q_2_ change to Q_1_’ and Q_2_’, respectively. Thus, according to positional changes of the light spot in the Z-axis direction on two QDs, the roll error $\varepsilon_{xx}$ of the linear axis is given as

(4a) $$\varepsilon_{xx}=\frac{\Delta Z_{QD1}-\Delta Z_{QD2}}{2h}$$

where, $\Delta Z_{QD1}$ and $\Delta Z_{QD2}$ are the positional changes in the Z-axis direction on QD1 and QD2, respectively. Both of them can be obtained by the system. h is the distance between beams 1 and 3, and it is a constant.

Similarly, the tilt motion error $\varepsilon_{xc}$ around the X-axis of the rotary axis can be calculated as

(4b) $$\varepsilon_{xc}=\frac{\Delta Z_{QD1}-\Delta Z_{QD2}}{2h}$$

### 4.5. Common-Path Compensation for Laser Beam Drift

Except for the positioning error of the linear axis and radial motion error in the X-axis direction of the rotary axis, the other 5DOF geometric motion error measurements are based on laser collimation. Beam drift, which is mainly caused by slow deformation of the mechanical structure and air turbulence, influences the accuracy of 5DOF geometric motion error measurements. Therefore, a common-path compensation method is integrated into the proposed system [26]. As shown in Figure 3e, if there is an angular drift of laser beam, the spot position of the beam on the PSD2 that is reflected by BS4 will change and the angular drift can be expressed as

(5) $$\begin{array}{l} \Delta \alpha =\frac{\Delta Y_{PSD2}}{f_{2}}\\ \Delta \beta =\frac{\Delta Z_{PSD2}}{f_{2}} \end{array}$$

where Δα and Δβ denote the beam’s angular drift around the Z- and Y-axis, respectively. $\Delta Y_{PSD2}$ and $\Delta Z_{PSD2}$ denote the positional changes on PSD2 in the Y- and Z-axis direction, respectively. Both of them can be obtained by the system. $f_{2}$ is the focal length of L2, and it is a constant.

In order to compensate for the measurement error caused by the laser drift, Equations (2a) and (3a) for computing the straightness errors, the pitch and yaw of the linear axis can be corrected as

(6a) $$\begin{array}{l} \delta_{yx}=\frac{\Delta X_{QD2}}{2}\pm L\Delta \alpha \\ \delta_{zx}=\frac{\Delta Z_{QD2}}{2}\pm L\Delta \beta \\ \varepsilon_{zx}=\frac{\Delta Y_{PSD1}}{2f_{1}}\pm \Delta \alpha \\ \varepsilon_{yx}=\frac{\Delta Z_{PSD1}}{2f_{1}}\pm \Delta \beta \end{array}$$

where “±” is decided by the real situation.

Similarly, Equations (2b) and (3b) for the rotary axis measurements can be corrected as

(6b) $$\begin{array}{l} \delta_{yc}=\frac{\Delta X_{QD2}}{2}\pm L\Delta \alpha \\ \delta_{zc}=\frac{\Delta Z_{QD2}}{2}\pm L\Delta \beta \\ \varepsilon_{zc}=\frac{\Delta Y_{PSD1}}{2f_{1}}+\theta_{ref}-\theta \pm \Delta \alpha \\ \varepsilon_{yc}=\frac{\Delta Z_{PSD1}}{2f_{1}}\pm \Delta \beta \end{array}$$

Theoretically, laser beam drift has no influence on the roll measurement of the linear axis and the tilt motion error measurement around the X-axis of the rotary axis. Thus, Equations (4a) and (4b) require no correction.

All the equations from Equations (1a)–(6b) are applicable for other axes of machine tools as well, including Y- and Z-axis, and the A-axis. 

## 5. Experimental Setup and Results

### 5.1. System Development

Based on the measurement principles mentioned above, an integrated system was developed, which consisted of two QDs (QP50-6SD2-DIAG, Pacific Silicon Sensor, resolution: 0.08 μm), two PSDs (DL16-7-PCBA3, First Sensor, resolution: 0.5 μm), two RRs (N-BK7, Edmund, accuracy: 3 arcsec), and other optical elements. Additionally, a commercial laser interferometer (MI5000, SIOS, resolution: 20 pm), which can measure up to 800 mm/s at a range of 5 m, was combined into the system. A reference rotary axis, which is consisted of a step-rotary table (MRS201, BOCI, resolution: 0.33 arcsec) and a circular-grating angle-encoder (RON886, Heidenhai, accuracy: 1.0 arcsec, resolution: 0.2 arcsec), was used for a rotary axis servo tracking measurement at a speed up to approximately 0.8 degrees per second.

### 5.2. Experimental Results of Calibration, Resolution, and Stability

A grating ruler (LG-50, accuracy: 0.1 μm, resolution: 50 nm) was used to calibrate the linear errors in Y- and Z-axis directions, and an autocollimator (Collapex EXP, accuracy: 0.2 arcsec, resolution: 0.01 arcsec) was used to calibrate the angular errors around Y- and Z-axes. The results are listed in the “Calibration” column in Table 2, and show that the proposed system has good linearity. For example, in the range of ±100 μm for measuring linear error in X-axis direction, the linear-fit determination coefficient (R^2^) is up to 0.9997 and the standard deviation is 0.81 μm. 

**Figure 4:**
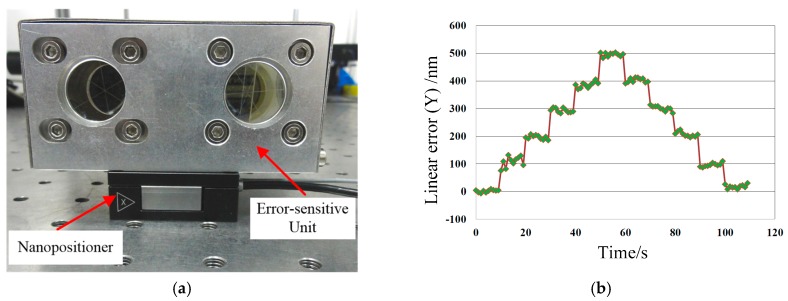
Resolution test: (**a**) Experimental setup; (**b**) Experimental results.

A piezo nanopositioner (P-611.1S, PI, resolution: 2 nm) was used to obtain the real measurement resolution of the linear errors in Y- and Z-axis directions of the proposed system. As shown in Figure 4a, the error-sensitive unit of the proposed system was fixed on the piezo nanopositioner, which was driven to move from 0 to 500 nm and back to the starting point at intervals of 100 nm. The measurement results were recorded automatically and are presented in Figure 4b, which shows that the measurement resolution is 100 nm. As the focal length of L1 and L2 is 200 mm and the distance between beams 1 and 3 is 30 mm, the measurement resolutions of angular errors can be calculated according to Equations (3a) and (4a). The results are listed in the “Resolution” column in Table 2.

The stability experiment was performed within 30 min, and the temperature changed 0.2 degrees. The error-sensitive unit was placed 300 mm away from the measurement unit. The experimental results were automatically recorded and the beam angular drift was compensated in real time by common-path compensation. The standard deviations of stability are listed in the “Stability” column in Table 2, and these demonstrate that the proposed system has good stability.

### 5.3. Experimental Results of the Linear Axis

**Figure 5:**
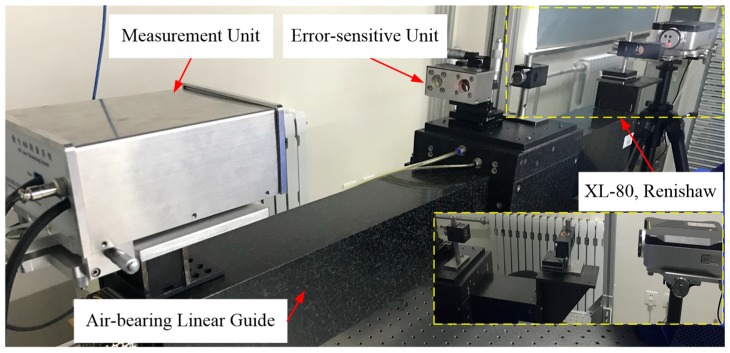
Measurement experiments for the linear axis.

As shown in Figure 5, the proposed system was used to simultaneously measure 6DOF geometric motion errors of an air-bearing linear guide. A laser interferometer (XL-80, Renishaw, linear resolution: 1 nm, angular resolution: 0.01 arcsec) was used for comparison. It is important to note that roll cannot be measured by XL-80 [30]. Thus, an electronic level (WL11, Qianshao, accuracy: 0.2 arcsec) was used for roll comparison. In the experiment, the air-bearing linear guide was driven at a speed of 5 mm/s, and the moving distance was 500 mm at intervals of 50 mm.

**Figure 6:**
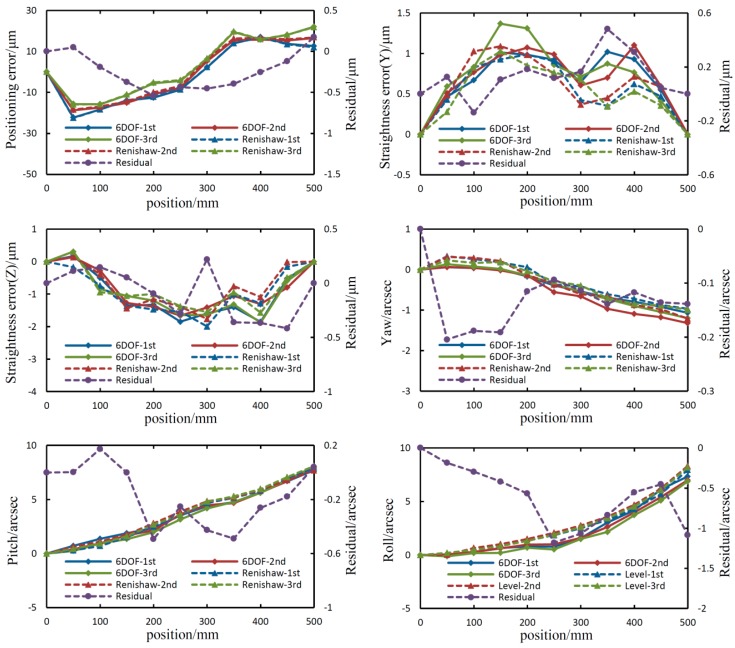
Results of repeatability tests and comparison experiments.

It took about 140 min to perform three repeated measurements using the proposed system and the standard instruments. The environmental parameters, including temperature, pressure, and humidity, were recorded by an environmental compensator (XC-80, Renishaw). It shows that the temperature of the laboratory was 24 ± 0.2 degrees, the relative humidity was 23.5 ± 1%, and the pressure was 1014.5 mbar. The results of the repeatability tests and comparison experiments are presented in Figure 6 and summarized in Table 3.

The experimental results in Figure 6 and Table 3 demonstrate a similar repeatability error between the proposed system and standard instruments. For example, the repeatability error for yaw measurement, which is calculated using the formula “±(maximum − minimum)/2”, is ±0.17 arcsec for the proposed system, and ±0.15 arcsec for the Renishaw interferometer. The primary reasons for the repeatability errors of the proposed system are the random error and laser beam drift—which cannot be completely compensated by the common-path beam drift measurement.

There are some comparison deviations between the proposed system and standard instruments. For instance, for the positioning error measurements, the maximum deviation between the mean of three measurements from the proposed system and that from the standard instrument is 0.54 μm. For comparison deviations between the proposed system and the standard instruments, the main reason is that their error-sensitive units are located at a different position, and it is almost impossible for them to measure the same point. Additionally, manufacturing and installation errors of optical elements and error crosstalk between 6DOF geometric motion errors also contribute to comparison deviation, especially for roll measurement. Theoretically, such deviation can be decreased by mathematical analysis and error modeling.

### 5.4. Experimental Results of the Rotary Axis

**Figure 7:**
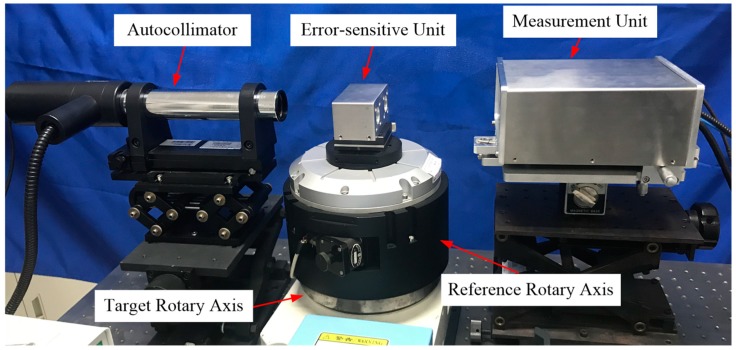
Measurement experiments of rotary axis.

As shown in Figure 7, an indexing table (HSD-200RT, accuracy < 60 arcsec, indexing angle: 30 degrees) was used as the target rotary axis. An autocollimator (Collapex EXP, accuracy: 0.2 arcsec, resolution: 0.01 arcsec) was used for the comparison. A plane mirror, which was fixed on the back of the error-sensitive unit of the proposed system, was used as the target of the autocollimator. Thus, the angular positioning error and tilt motion error around the Y-axis can be measured by the two systems simultaneously. The distance between the measurement unit and the error-sensitive unit was 250 mm. In the experiment, the rotation speed of the target rotary axis was set as 0.55 degrees per second.

**Figure 8:**
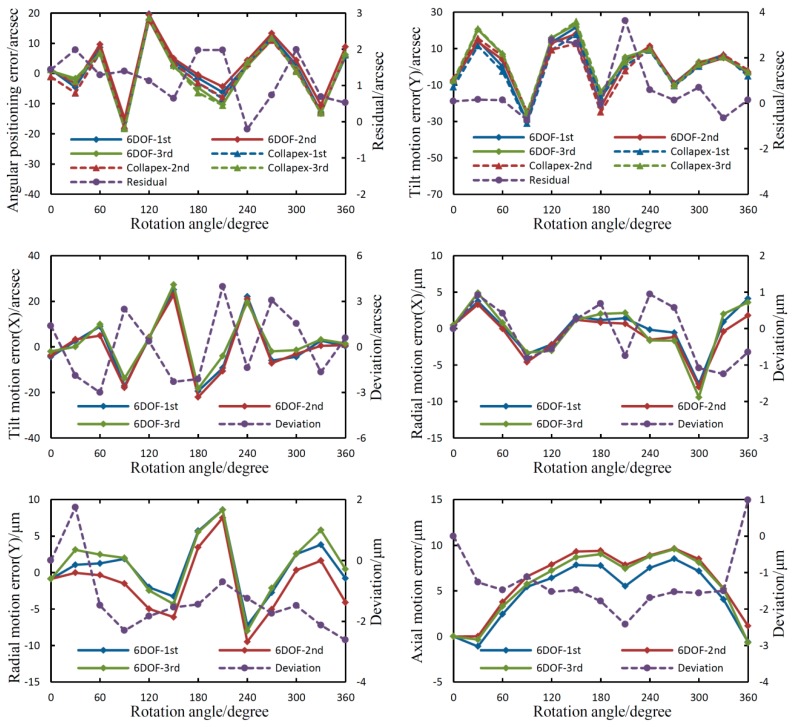
Results of repeatability tests and comparison experiments.

The measurement was repeated three times within 45 min, and the results of the repeatability tests and comparison experiments are presented in Figure 8.

As shown in Figure 8, the repeatability error for the angular positioning error and tilt motion error around the Y-axis are ±3.3 and ±4.3 arcsec for the proposed system, respectively; and ±2.6 and ±6.5 arcsec for the autocollimator, respectively. The maximum deviations are 2.0 arcsec for the angular positioning error, and 3.6 arcsec for tilt motion error around the Y-axis. The repeatability error for the tilt motion error around the X-axis is ±4.0 arcsec; those of the radial motion error in the X- and Y-axis direction are ±1.2 and ±2.6 μm respectively; that of the axial error motion is ±2.4 μm.

The repeatability errors of 6DOF geometric motion errors for the rotary axis are larger than those of the linear axis. This is because the target rotary axis itself demonstrates poor repeatability according to the results from the autocollimator. It should be noted that laser beam drift has a great influence on the linear axis measurements, and the manufacturing and installation accuracy of the reference axis has great influence on the rotary axis measurements. Generally speaking, the errors of the proposed 6DOF system mainly include the random error, the manufacturing and installation errors, and the error crosstalk, they are very complex and cannot be given in detail in this paper due to the limitation of length. We will analyze these errors and establish a compensation model in future works.

## 6. Conclusions

It is the primary approach and developmental direction to improve and maintain the machining accuracy of CNC machine tools by simultaneously measuring the multi-DOF geometric motion errors of the linear and rotary axes, and subsequently establishing an error compensation model. In this study, based on a combination of laser interferometry and laser collimation, we proposed a direct measurement method for simultaneously measuring the 6DOF geometric motion errors for linear and rotary axes, and developed a corresponding measurement system. The feasibility and effectiveness of the method and system were verified through a series of experiments. Future work will focus on error modeling and accuracy improvement.

## 7. Patents

The authors also published a USA patent No. US 9857161 B2 resulting from the work reported in this manuscript.

## Figures and Tables

**Table 1:** Six-degrees-of-freedom (6DOF) geometric motion error definitions.

	Translational Errors			Angular Errors		
	Linear Error in X-axis Direction	Linear Error in Y-axis Direction	Linear Error in Z-axis Direction	Angular Error around X-axis	Angular Error around Y-axis	Angular Error around Z-axis
Linear axis (X)	$\delta_{xx}$	$\delta_{yx}$	$\delta_{zx}$	$\varepsilon_{xx}$	$\varepsilon_{yx}$	$\varepsilon_{zx}$
Rotary axis (C)	$\delta_{xc}$	$\delta_{yc}$	$\delta_{zc}$	$\varepsilon_{xc}$	$\varepsilon_{yc}$	$\varepsilon_{zc}$

**Table 2:** Experimental results of calibration, resolution, and stability.

Geometric Motion Error	Calibration			Resolution	Stability
	Measurement Range	Standard Deviation	R^2^	Resolution	Standard Deviation
Linear error in X-axis direction	5 m	-	-	20 pm	0.04 um
Linear error in Y-axis direction	± 100 μm	0.81 μm	0.9997	100 nm	0.07 μm
Linear error in Z-axis direction	± 100 μm	0.81 μm	0.9997	100 nm	0.09 um
Angular error around X-axis	± 680 arcsec	-	-	0.69 arcsec	0.45 arcsec
Angular error around Y-axis	± 200 arcsec	0.98 arcsec	1.0	0.26 arcsec	0.21 arcsec
Angular error around Z-axis	± 200 arcsec	0.98 arcsec	1.0	0.26 arcsec	0.16 arcsec

**Table 3:** Performance of the 6DOF system.

Parameter	Repeatability Error		Maximum Comparison Deviation
	Proposed System	Standard Instruments	
Positioning error/μm	-	-	0.54
Straightness error (Y)/μm	±0.25	±0.19	0.48
Straightness error (Z)/μm	±0.37	±0.30	0.42
Yaw/arcsec	±0.17	±0.15	0.20
Pitch/arcsec	±0.30	±0.21	0.49
Roll/arcsec	±0.60	±0.29	1.18
